# The immunohistochemical evaluation of selected markers in the left atrium of dogs with end-stage dilated cardiomyopathy and myxomatous mitral valve disease – a preliminary study

**DOI:** 10.1186/s13620-016-0077-2

**Published:** 2016-12-01

**Authors:** Izabela Janus, Małgorzata Kandefer-Gola, Rafał Ciaputa, Agnieszka Noszczyk-Nowak, Urszula Pasławska, Massimiliano Tursi, Marcin Nowak

**Affiliations:** 1 Division of Pathomorphology and Veterinary Forensics, Department of Pathology, Wroclaw University of Environmental and Life Sciences, Wroclaw, 50375 Poland; 2 Department of Internal Medicine and Clinic of Diseases of Horses, Dogs and Cats, Wroclaw University of Environmental and Life Sciences, Wroclaw, 50366 Poland; 3 Department of Animal Sciences, University of Turin, Grugliasco, TO Italy

**Keywords:** Dilated cardiomyopathy, Myxomatous mitral valve disease, DCM, MMVD, Immuniohistochemistry, Left atrium

## Abstract

**Background:**

Dilated cardiomyopathy (DCM) and myxomatous mitral valve disease (MMVD) are the most common diseases noted in dogs. Although their pathogenesis varies, both include a significant enlargement of the left atrium.

The study was carried out on left atrial specimens obtained from 56 dogs, including those from 34 dogs with clinically diagnosed MMVD, 15 dogs with DCM and 7 dogs without heart disease (control group). Dogs in the MMVD and the DCM groups presented with left atrial enlargement and stage D heart failure. The specimens underwent immunohistochemical examination using desmin, vimentin, periostin and caspase-3 antibodies.

**Results:**

There were alterations in the expression of the studied proteins in the study groups compared to the control group. The changes included: irregularity of desmin cross-striation and desmosomes, a higher amount of vimentin-positive cells, a change in the periostin expression pattern from cytoplasmic to extracellular, and a lower expression of caspase-3. The alterations were more pronounced in the DCM group than in the MMVD group.

**Conclusions:**

During heart failure, the pattern of desmin, vimentin, periostin and caspase-3 expression alters in the left atrium, regardless of the cause. The changes are more pronounced in dogs with DCM than in dogs with MMVD and similar left atrial enlargement, suggesting that volume overload may not be the only cause of myocardial changes in DCM.

## Background

Dilated cardiomyopathy (DCM) is a disease of various aetiologies diagnosed mainly in large and giant breed dogs. It is characterised by an enlargement of all the heart chambers without other concurrent heart diseases [1–6].

Myxomatous mitral valve disease (MMVD) involves a progressing degeneration of the valve leaflets resulting in mitral regurgitation and left atrial and ventricular enlargement [7].

The majority of research concerning heart failure resulting from these diseases focuses on changes occurring in the ventricles. However, current research suggests that myocardial remodelling is more pronounced in the left atrium than the left ventricle [8, 9].

Although both DCM and MMVD are accompanied by a considerable enlargement of the left atrium (LA), our previous study [10] showed significant differences in the remodelling of the LA wall in these two diseases. DCM is accompanied by a more pronounced interstitial fibrosis with less distinct perivascular fibrosis and myocardial arterial narrowing than MMVD. Additionally, the cardiomyocyte degenerative changes are more severe in DCM than in MMVD. Hence, we decided that it might be useful to assess and compare the expression of various cell markers of cardiomyocyte and interstitial tissue in these diseases. Since myocardial remodelling affects not only cardiomyocytes but also the interstitial tissue, we assessed various cell markers: desmin as a standard cardiomyocyte marker, vimentin as a typical cell marker expressed in mesenchymal cells, periostin as a novel marker for myocardial remodelling, and caspase-3 as a marker of cell apoptosis. This preliminary study is the first step toward extensive research on a wide variety of cell markers.

Desmin is a protein that builds intercalated discs. It is the main component of the cardiomyocyte cytoskeleton and forms the intermediate filament. Due to its role in the formation of striation within the myocardium, desmin plays an important part in the normal functioning of the myocardium by forming striation. In the course of heart failure, the structure of the cardiomyocyte is compromised and there is a disorganisation of desmin filaments and loss of cross striation [11–14].

Vimentin builds intermediate filaments of mesenchymal cells and is produced in fibroblasts, macrophages, endothelial cells and smooth muscle cells. In normal cardiac tissue, vimentin-positive cells are spread among cardiomyocytes, forming a delicate stroma, the amount of which increases with progressing heart failure [12, 14].

Periostin expression is particularly high in collagen-rich connective tissue subjected to mechanical stress e.g. heart valves [15]. In a normal heart, periostin plays a role in the morphogenesis of heart valves. It is also produced by fibroblasts and ventricular cardiomyocytes during heart failure and over the past few years has served as a marker of myocardial remodelling in volume overload in humans and as a potential new target for treatment possibilities [16–18].

In quiescent cells, caspases exist as inactive zymogens that are readily activated by autocatalytic processes or by other caspases following a death signal [19]. The activation of caspase-group proteins is crucial during apoptosis, which is thought to be one of the factors causing progressing heart failure of various aetiologies [20, 21].

The objectives of the study were: (1) the assessment and comparison of the expression of desmin, vimentin, periostin and caspase-3 in the left atrial myocardium in dogs with DCM and MMVD showing severe left atrial enlargement; (2) comparison of those results with the results from a group of dogs without heart disease; (3) the evaluation of the correlation between the expression of the examined proteins and heart remodelling based on the histological examination.

## Methods

### Population study and inclusion criteria

The study was conducted on left atrial specimens obtained from 56 dogs divided into 3 groups: dogs presenting with dilated cardiomyopathy (DCM group; *n* = 15), dogs with left atrial enlargement due to myxomatous mitral valve disease (MMVD group; *n* = 34) and dogs that underwent euthanasia due to causes not related to cardiovascular system and that did not show intravital or post-mortem features of cardiorespiratory disease and LA enlargement (control group; *n* = 7).

Dogs met the inclusion criteria if they had an enlarged left atrium as defined by an echocardiographic examination (left atrial (LA)-to-aortic root (Ao) diameter ratio; LA/Ao >1.7 [22]), which was confirmed in the post-mortem examination. A dilation of the left ventricle and a decreased fractional shortening (*FS* < 20%), both noted during the echocardiographic examination [3, 5, 6], with normal or type 1 mitral valve lesions (according to Whitney [23]) were required to classify dogs in the DCM group. Dogs in the MMVD group were required to have a preserved left ventricular systolic function (*FS* > 20%, noted by echocardiographic examination) with type 3 or 4 mitral valve lesions (according to Whitney [23]). At the time of death or euthanasia, all the dogs in the DCM and MMVD groups presented with stage D heart failure (according to the ACVIM Consensus Statement [24]).

### Clinical and post-mortem examination

Fourty two dogs underwent a standard clinical and cardiological examination with ECG, an echocardiographic examination and 24-h ECG (Holter) monitoring in cases where rhythm disturbances were suspected as described in our previous study [10]. After the examination, dogs in the DCM and the MMVD group underwent appropriate treatment to reduce the effects of progressive heart failure and/or arrhythmias, and were checked every 6 months or more frequently if necessary until the moment of death or euthanasia. All the dogs in the DCM and the MMVD group died or were euthanized within 5 years of diagnosis.

In 14 cases, some or all the results of the cardiac examination were not available. In those cases, a particular emphasis was put on the post-mortem examination to confirm the inclusion of the dogs in the DCM, MMVD, or control group.

The results of the echocardiographic examination (left atrial and left ventricular enlargement) were confirmed during the post-mortem examination, and the type of mitral valve lesion was assessed. Heart measurements were taken using a manual 150 mm Beerendonk caliper, accurate to the nearest 1/20 mm. The measurements were taken in planes compatible with those used during the echocardiographic examination. The left atrial diameter was related to the diameter of the aorta (as in LA/Ao ratio) and the left ventricular internal diameter and wall thickness were measured beneath the mitral valve with the omission of the papillary muscles. The type of mitral valve lesion according to Whitney [23] was determined based on the thickness of the valve leaflets and tendinous chords. Specimens from the left atrial wall were collected from each dog for further histopathological and immunohistochemical analysis. The specimens were collected in the dorso-ventral plane from the middle portion of the atrial free wall, at the same site in all the dogs.

### Histopathological examination

The specimens were fixed in 7% buffered formalin, dehydrated, embedded in paraffin blocks and sectioned at 4 μm. Each specimen was randomly assigned a number to allow a blinded examination. The specimens were stained with haematoxylin-eosin and Masson-Goldner trichrome and evaluated for interstitial and perivascular fibrosis, cardiomyocyte degeneration, intramyocardial arterial narrowing and inflammatory infiltrates as described in our previous study [10]. The interstitial fibrosis was evaluated after superimposing a 16-field grid over the digital image and counting the number of fields that showed the presence of fibrosis [4, 10]. The severity of perivascular fibrosis was scored from 0 (no fibrosis) to 3 (severe fibrosis) [10]. Cardiomyocyte degenerative changes (abnormal cell nuclei, loss of striation and changes in the structure of the cardiomyocytes) were scored from 0 (normal myocardium or changes affecting <25% of myofibres) to 3 (changes affecting >75% of myofibres) [10]. Intramyocardial arterial narrowing was assessed using the lumen area ratio (LAR; the luminal area of the vessel divided by the total vessel area, not including the adventitia) [10, 25]. Inflammatory infiltrates were evaluated according to the number of inflammatory cells in a high power field [10]. Each of the features was evaluated in 20 randomly chosen fields per slide (for interstitial fibrosis, cardiomyocyte degeneration and inflammatory infiltrates) or in at least 10 vessels per slide (for perivascular fibrosis and intramyocardial arterial narrowing). An average score was counted for each feature in every specimen.

### Immunohistochemical examination

The paraffin sections were placed on silanized microscope slides and underwent a standardised immunohistochemical staining procedure using Autostainer Link48 (DakoCytomation, Glostrup, Denmark). First, deparaffinization, rehydration and antigen retrieval was performed using EnVision FLEX Target Retrieval Solution (97 °C, 20 min) in PT-Link. Activity of endogenous peroxidase was blocked by 5 min incubation with EnVision FLEX Peroxidase-Blocking Reagent (DakoCytomation). The sections were overlayed with primary antibodies, including a mouse monoclonal anti-desmin-clone D33 (DAKO, Danmark); a mouse monoclonal anti-vimentin-clone V9 (DAKO, Danmark); rabbit polyclonal anti-periostin (Abcam, UK) and a mouse monoclonal anti-caspase-3 – clone 3CSP03 (Abcam, UK) for 20 min. No secondary antibodies were used.

Then slides were incubated with EnVision FLEX/HRP (20 min). 3,3′-diaminobenzidine (DAB, Dako) was utilized as the peroxidase substrate and the sections were incubated for 10 min. Finally, all sections were counterstained with EnVision FLEX Hematoxylin (Dako) for 5 min. After dehydration in graded ethanol concentrations (70%, 96%, 99,8%) and in xylene, slides were closed with coverslips in Dako Mounting Medium (DakoCytomation). Specimens from the control group collected for the analysis of the expression of desmin, vimentin and caspase-3 served as positive controls. Specimens of heart valves stained according to the abovementioned protocol were used as positive controls for the expression of periostin; sections immunostained in the absence of a primary antibody were used as negative controls.

Photomicrographs of the examined tissues were subjected to computer-assisted image analysis, using a computer coupled to an optical Olympus BX53 microscope, equipped with an Olympus Color View IIIu digital camera (Olympus, Japan) and cell^A software (Olympus Soft Imaging Solution GmbH, Germany).

Since in the literature the immunohistochemical expression of the examined markers in the myocardium is reported mainly descriptively [11, 14, 26], we evaluated the pattern of expression for each cell marker. Moreover, to allow a semi-quantitative comparison of the examined markers and their correlation with myocardial remodelling, their expression in cardiomyocyte and interstitial tissue was appraised in 10 randomly chosen high power fields using a three step approach: 1) the intensity of expression (scored from 0–no expression, to 3–strong expression), 2) the percentage of marker-positive cells within a high power field, 3) the modified semiquantitative immune-reactive score (IRS) scale according to Remmele [27]. The latter takes into account both the intensity of the expression and the percentage of positive cells. Additionally, the expression of desmin within the desmosomes was scored from 0 (no expression) to 3 (strong expression). An average score from 10 examined fields was counted for each feature.

### Statistical analysis

The normality of the data was evaluated using Shapiro-Wilk analysis. The obtained data underwent statistical analysis using the Kruskal-Wallis rang analysis and Spearman’s correlation test using StatisticaPL for Windows (StatSoft, Poland). Statistical significance was set at *p* ≤ 0.05.

## Results

The DCM group consisted of 13 males and 2 females, aged from 4 to 11 years (median 8 years), weighing from 30 to 45 kg (median 31 kg), and included eight Doberman Pinchers, four German Shepherds, two boxers and one Great Dane. The MMVD group consisted of 21 males and 13 females, aged from 8 to 19 years (median 14 years), weighing from 5 to 35 kg (median 10.5 kg), and included 23 mixed-breed dogs, five dachshunds, two miniature Pinchers and one dog of each breed: Black Russian Terrier, Shar-Pei, Cairn Terrier and the American Staffordshire Terrier. The control group consisted of 5 males and 2 females, aged from 1 to 12 years (median 10 years), weighing from 5 to 25 kg (median 5 kg), including three mixed-breed dogs and one dog of each breed: miniature Schnauzer, German Shepherd, dachshund, boxer.

There was no significant difference between the LA/Ao ratio in both examined groups in either the echocardiographic or post-mortem examination (*p* > 0.05).

### Immunohistochemical examination

**Fig. 1:**
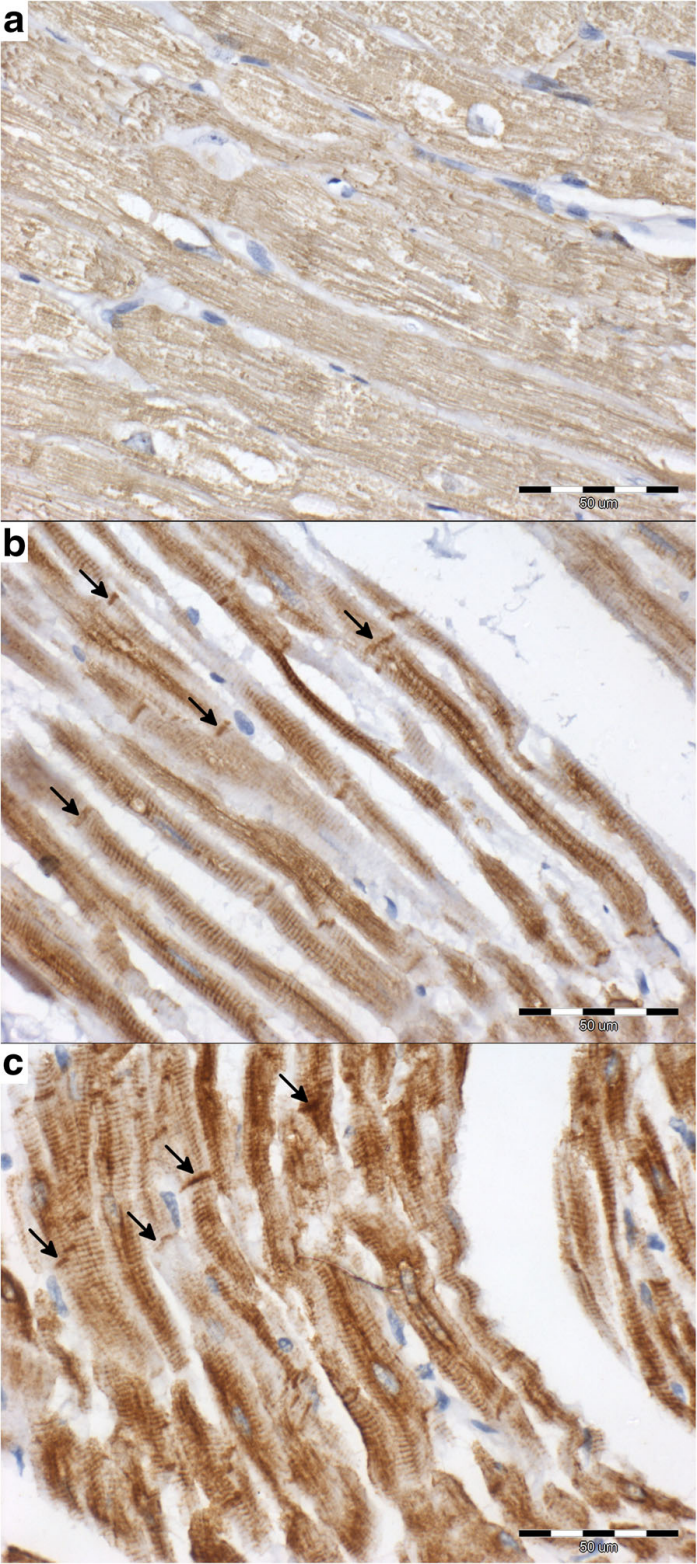
The cytoplasmic expression of desmin in cardiomyocyte (*brown*) in examined groups (interstitial tissue stained *blue*; scale ba*r* = 50 μm). **a** – DCM group: the cardiomyocytes are swollen and show irregular desmin expression scored 1 with no visible cross-striation; the desmosomes are not visible (score 0); **b** – MMVD group: cardiomyocyte with regular, well-preserved striation; the intensity of desmin expression scored 3; desmosomes are well visible (*arrows*; score 3); **c** – control group: cardiomyocyte with regular, well-preserved striation; the intensity of desmin expression scored 3; desmosomes are well visible (*arrows*; score 3)

**Fig. 2:**
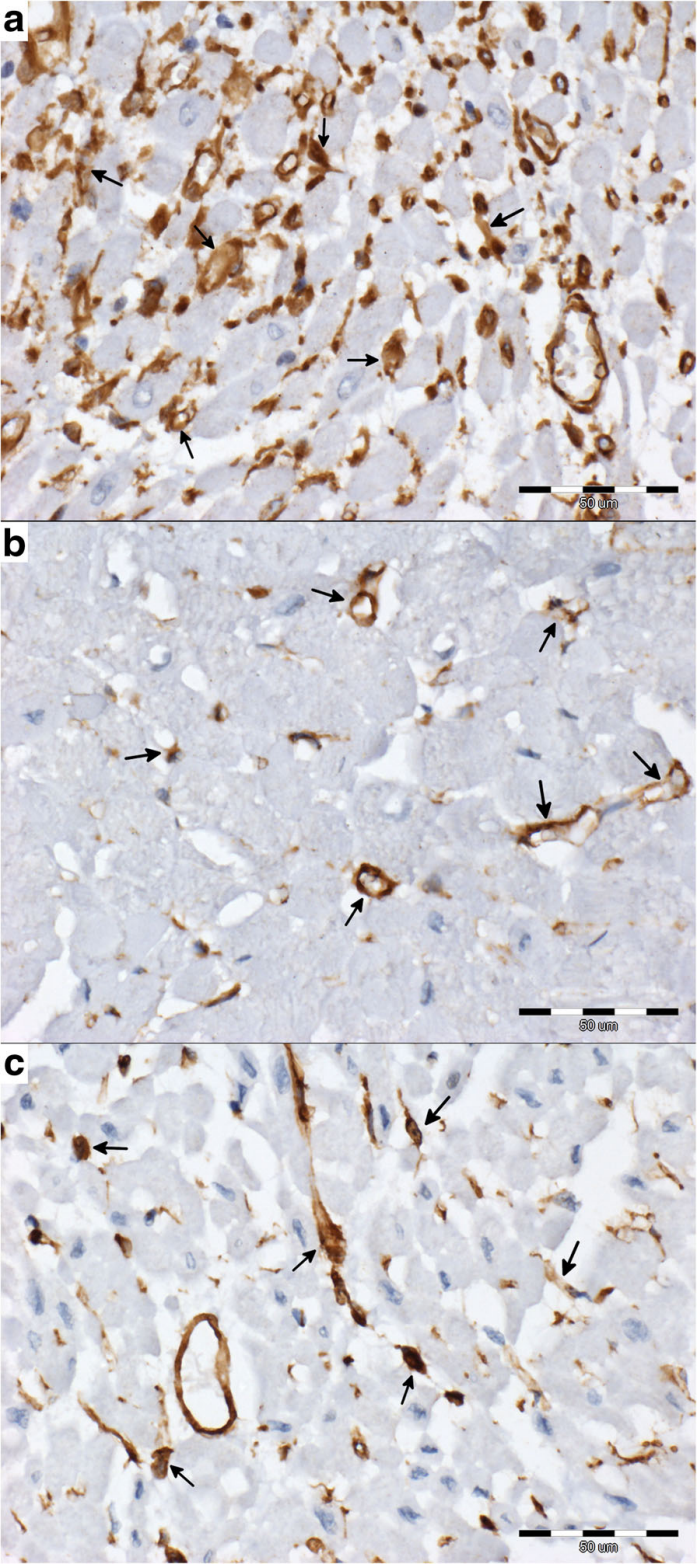
The cytoplasmic expression of vimentin (*brown*; arrows) in interstitial tissue in examined groups (vimentin-negative cells stained *blue*; scale ba*r* = 50 μm). **a** – DCM group: abundant vimentin-positive cells between cardiomyocyte; the vimentin-positive cells show staining intensity scored 3; **b** – MMVD group: sparse vimentin-positive cells between cardiomyocyte; the vimentin-positive cells show staining intensity scored 3; **c** – control group: sparse vimentin-positive cells between cardiomyocyte; the vimentin-positive cells show staining intensity scored 3

**Fig. 3:**
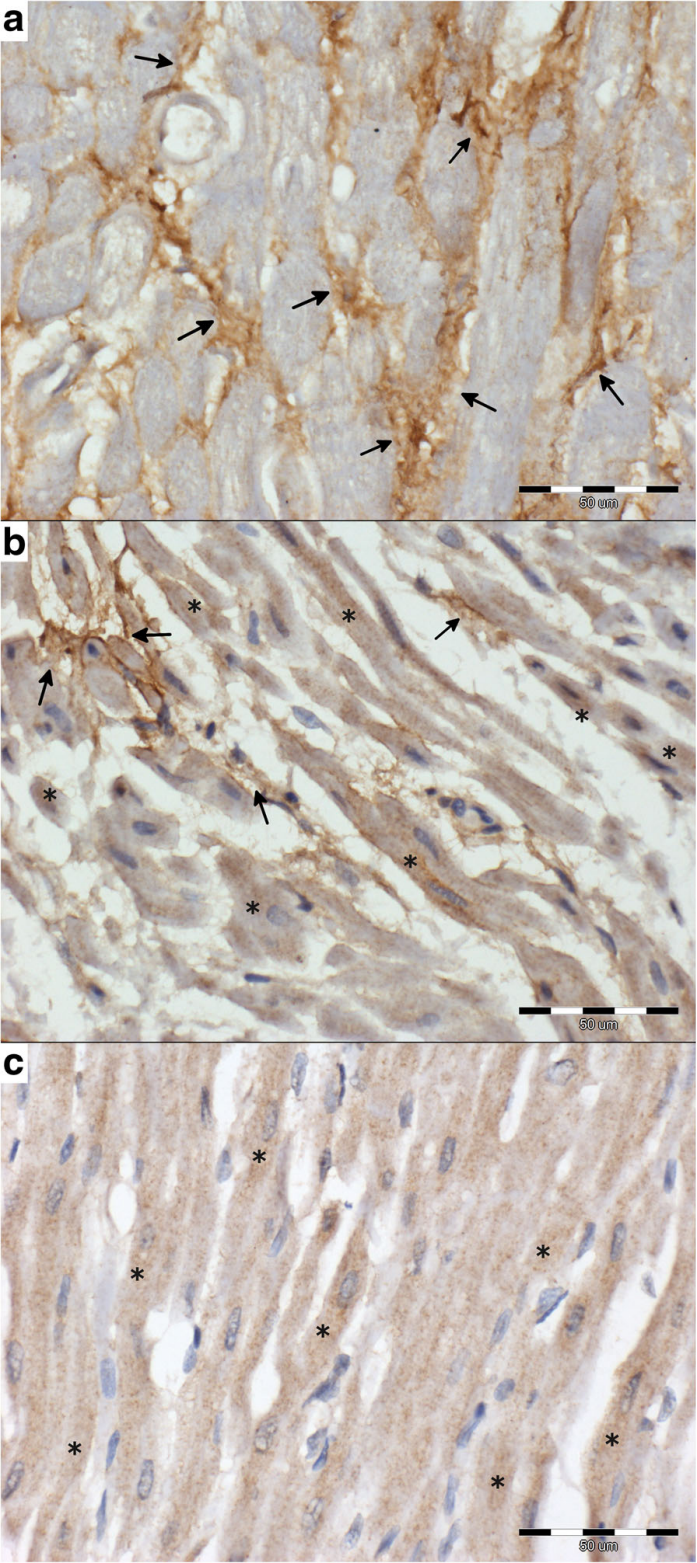
The cytoplasmic expression of periostin (*brown*) in examined groups (periostin-negative cells stained *blue*; scale ba*r* = 50 μm). **a** – DCM group: strong expression in interstitial tissue (*arrows*) with intensity scored 2; **b** – MMVD group: diffused cytoplasmic expression in cardiomyocyte (*) with mild expression in interstitial tissue (*arrows*); intensity of expression scored 1; **c** – control group: diffused cytoplasmic expression in cardiomyocyte (*); intensity of expression scored 1

**Fig. 4:**
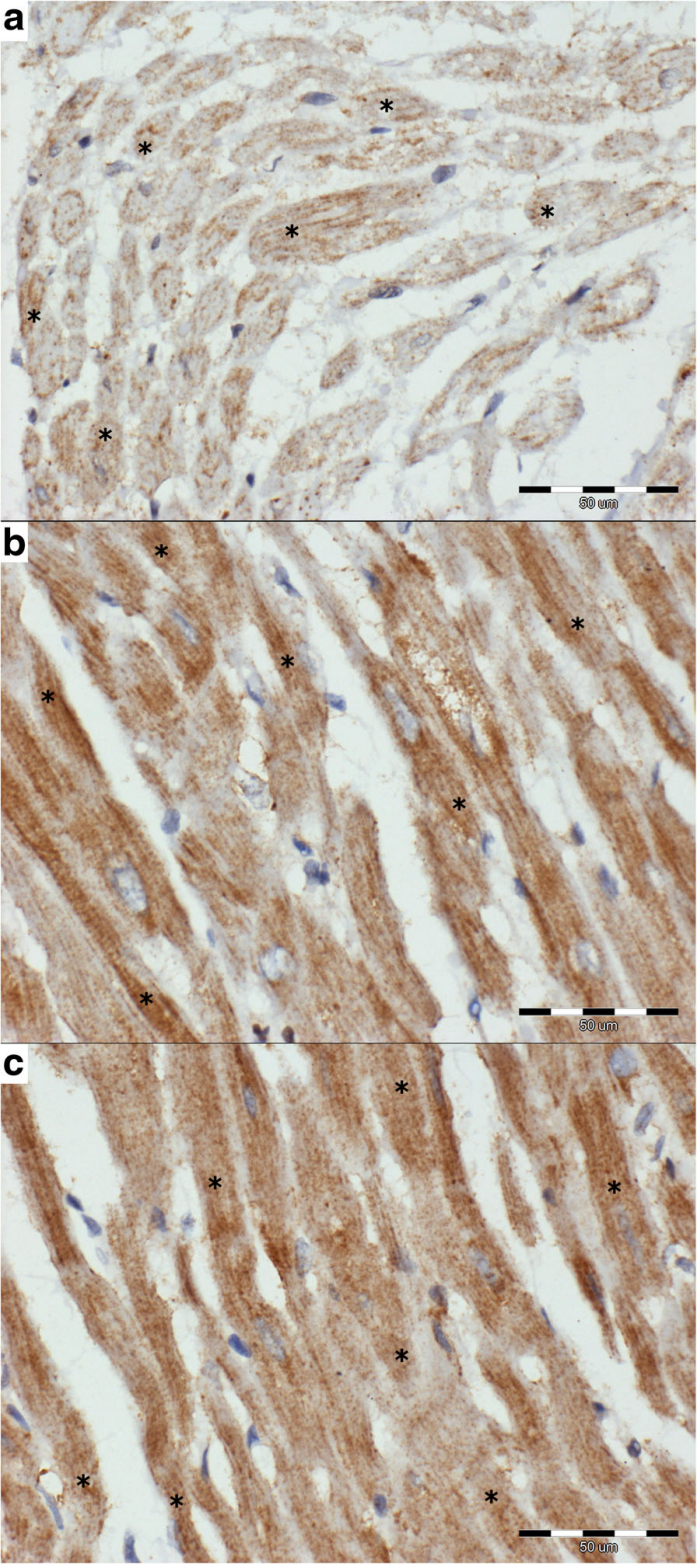
The cytoplasmic expression of caspase-3 (*brown*) in examined groups (caspase-3-negative cells stained *blue*; scale ba*r* = 50 μm). **a** – DCM group: diffuse mild cytoplasmic expression in cardiomyocyte (*) scored 1; approximately 60% of cardiomyocyte showing positive expression; **b** – MMVD group: diffuse strong cytoplasmic expression in cardiomyocyte (*) scored 2; over 90% of cardiomyocyte show positive expression; **c** – control group: diffuse strong cytoplasmic expression in cardiomyocyte (*) scored 2; over 90% of cardiomyocyte show positive expression

The results of the protein expression in each group of dogs are presented in Tables 1 and 2 and Figs. 1, 2, 3 and 4

**Table 1:** The results of immunohistochemical examination in studied dogs

Examined feature	DCM	MMVD	control	*p*-value
DES intensity; median (range)	1 (1–2)^1^	2 (1–3)	2 (2–3)^1^	^1^ 0.006
DES %; average ± SD	85.8 ± 10.9	84.8 ± 9.3	82.2 ± 8.6	
DES Remmele; median (range)	4 (3–8)^1, 2^	6 (4–9) ^1^	7 (6–10)^2^	^1^ 0.03
				^2^ 0.001
DES desmosomes; median (range)	0 (0–3)^1^	2 (0–3)	2 (1–3)^1^	^1^ 0.02
VIM intensity; median (range)	3 (2–3)	3 (2–3)	2 (2–3)	
VIM %; average ± SD	15.3 ± 6.3^1,2^	10.99 ± 5.7^1^	10.5 ± 1.97^2^	^1^ 0.03
				^2^0.02
VIM Remmele; median (range)	4 (2–6)	3 (2–6)	4 (3–5)	
PER intensity; median (range)	1 (0–2)	1 (1–2)	1 (1–1)	
PER %; average ± SD	27.96 ± 27.6^1^	46.4 ± 28.5	67.76 ± 9.7^1^	^1^ 0.03
PER Remmele; median (range)	2 (0–3)	3 (1–4)	3 (3–3)	
CAS intensity; median (range)	1 (0–2)	1 (1–2)	1 (1–2)	
CAS %; average ± SD	46.9 ± 25.1^1,2^	73.6 ± 24.9^1^	84.6 ± 8.9^2^	^1^ 0.01
				^2^ 0.01
CAS Remmele; median (range)	3 (1–4)^1,2^	4 (1–7)^1^	4 (3–9)^2^	^1^ 0.01
				^2^ 0.01

*DES* desmin, *VIM* vimentin, *PER* periostin, *CAS* caspase, *SD* standard deviation, *DCM* dogs with dilated cardiomyopathy, *MMVD* dogs with myxomatous mitral valve disease; superscripts (^1,2^) indicate values showing significant statistical difference.

All the cell markers were evaluated for: the intensity of expression (scored from 0: no expression to 3: strong expression), the percentage of cells showing positive expression, as well as the Remmele score (scored from 0 to 12, taking into account both the intensity of expression and percentage of positive cells). Additionally the cardiomyocyte desmosomes were evaluated for desmin expression from 0 (no visible desmosomes) to 3 (well visible desmosomes)

**Table 2:** Type of the expression of cell markers in examined specimens in each group

Antibody	Pattern of expression	DCM (*n*; %)	MMVD (n; %)	Control (*n*; %)
DES	Regular, well-preserved striation	3 (20%)	19 (55.9%)	7 (100%)
	Irregular striation	9 (60%)	8 (23.5%)	0
	No expression	3 (20%)	7 (20.6%)	0
DES (desm)	Well-visible	3 (20%)	12 (35.3%)	7 (100%)
	Faint	9 (60%)	14 (41.2%)	0
	No expression	3 (20%)	8 (23.5%)	0
VIM	Abundant between cardiomyocytes	5 (33.3%)	2 (5.9%)	1 (14.3%)
	Sparse between cardiomyocytes	4 (26.7%)	26 (76.5%)	6 (85.7%)
	No expression	6 (40%)	6 (17.6%)	0
PER	Diffused cytoplasmic expression in cardiomyocytes with mild expression in interstitial tissue	5 (33.3%)	15 (44.1%)	5 (71.4%)
	Strong expression in interstitial tissue with mild cytoplasmic expression in cardiomyocytes	5 (33.3%)	10 (29.4%)	0
	No expression	5 (33.3%)	9 (26.5%)	2 (28.6%)
CAS	Diffuse strong cytoplasmic expression	4 (26.7%)	25 (73.5%)	7 (100%)
	Diffuse mild cytoplasmic expression	10 (66.7%)	6 (17.6%)	0
	No expression	1 (6.7%)	3 (8.8%)	0

*DES* desmin, *desm* desmosomes, *VIM* vimentin, *PER* periostin, *CAS* caspase, *DCM* dogs with dilated cardiomyopathy, *MMVD* dogs with myxomatous mitral valve disease

Desmin showed positive expression within the cardiomyocyte and was evaluated for the regularity and visibility of the cardiomyocyte cross-striation and for the visibility of desmosomes within the cardiomyocyte; vimentin showed positive expression within the interstitial tissue and no expression within the cardiomyocyte; periostin showed positive expression in both cardiomyocyte and interstitial tissue with variable intensity; caspase-3 showed positive expression only within the cardiomyocyte. All the cell markers showed cytoplasmic expression

### Desmin

In the normal myocardium, both striation and desmosomes are well visible. In enlarged left atria, the striation becomes irregular, unclear and the desmosomes are faint or do not stain. The changes in the desmin staining pattern are more pronounced in the DCM dogs than in the MMVD dogs with significantly lower staining intensity and lower Remmele scores in the first group (Tables 1 and 2, Fig. 1).

The intensity of the desmin expression showed a negative correlation with the enlargement of the cardiomyocyte nuclei (*p* < 0.05, *r* = −0.53), loss of cardiomyocyte striation (*p* < 0.05, *r* = −0.48) and cardiomyocyte degeneration (*p* < 0.05, *r* = −0.53). The percentage of desmin-positive cells showed a positive correlation with the enlargement of the cardiomyocyte nuclei (*p* < 0.05, *r* = 0.37), loss of cardiomyocyte striation (*p* < 0.05, *r* = 0.49) and cardiomyocyte degeneration (*p* < 0.05, *r* = 0.46). The desmin Remmele score showed a negative correlation with the enlargement of the cardiomyocyte nuclei (*p* < 0.05, *r* = −0.44), loss of cardiomyocyte striation (*p* < 0.05, *r* = −0.38) and cardiomyocyte degeneration (*p* < 0.05, *r* = −0.45). The desmosome desmin score showed a negative correlation with the enlargement of the cariomyocyte nuclei (*p* < 0.05, *r* = −0.5), loss of cardiomyocyte striation (*p* < 0.05, *r* = −0.44), cardiomyocyte degeneration (*p* < 0.05, *r* = −0.53) and a positive correlation with myocardial arterial narrowing (*p* < 0.05, *r* = 0.38).

### Vimentin

In all the examined specimens, vimentin was expressed within the interstitial tissue. The amount of vimentin-positive cells differed between the examined groups. We observed no difference in the intensity of the vimentin staining in all the groups. A significantly higher percentage of vimentin-positive cells was characteristic of the DCM group as compared to the remaining two groups (Tables 1 and 2, Fig. 2).

The percentage of vimentin-positive cells showed a negative correlation with perivascular fibrosis (*p* < 0.05, *r* = −0.39), a positive correlation with the enlargement of cardiomyocyte nuclei (*p* < 0.05, *r* = 0.34), and a negative correlation with arterial narrowing (*p* < 0.05, *r* = −0.37). The vimentin Remmele score showed a negative correlation with perivascular fibrosis (*p* < 0.05, *r* = −0.33) and with arterial narrowing (*p* < 0.05, *r* = −0.39).

### Periostin

Periostin was expressed in the normal myocardium, mainly in the cytoplasm of cardiomyocytes. There was also mild expression in the interstitial tissue. 29.4% of the MMVD specimens and 33.3% of the DCM specimens showed a strong expression of periostin in the interstitial tissue and only mild expression in the cardiomyocyte cytoplasm. Approximately 30% of the specimens in each group showed no expression of periostin. At the same time, significantly fewer periostin-positive cells were observed in the DCM group as compared to the remaining two groups (Tables 1 and 2, Fig. 3).

Both the percentage of periostin-positive cells and the periostin Remmele score showed a negative correlation with the enlargement of the cardiomyocyte nuclei (*p* < 0.05, *r* = −0.3).

### Caspase-3

Caspase-3 gave a strong diffuse cytoplasmic expression in all the specimens from the control group. A similar expression was observed in 73.5% of dogs in the MMVD group and in only 26.7% of dogs in the DCM group. The DCM group showed a significantly lower percentage of caspase-3-positive cells and a lower Remmele score as compared to the remaining two groups (Tables 1 and 2, Fig. 4).

The intensity of the expression of caspase-3 showed a negative correlation with the loss of striation (*p* < 0.05, *r* = −0.28) and a positive correlation with the arterial narrowing (*p* < 0.05, *r* = 0.51). Both the percentage of caspase-3-positive cells and caspase-3 Remmele score showed a negative correlation with the enlargement of cardiomyocyte nuclei (*p* < 0.05, *r* = −0.44 and *r* = −0.47, respectively), loss of striation (*p* < 0.05, *r* = −0.38 and *r* = −0.48, respectively) and cardiomyocyte degeneration (*p* < 0.05, *r* = −0.36 and *r* = −0.43, respectively).

## Discussion

Our study shows that although DCM and MMVD are both accompanied by LA enlargement, there are various changes occurring in the myocardium in the course of these diseases, with more pronounced changes in cardiomyocytes and interstitial tissue in DCM than MMVD.

During the progression of heart failure, the cross striation and desmosomes became less organised. The amount of interstitial tissue comprised of vimentin-positive cells increased. These changes were more pronounced in the DCM group than in the MMVD group. With heart failure, the structure of cardiomyocytes and intercalated disks undergoes disorganisation, which leads to loss of myofilaments and their supporting proteins, e.g. desmin [14, 26]. Cardiomyocyte remodelling is accompanied by the expansion of interstitial tissue with fibrosis and scar formation [26]. The interstitial tissue in failing hearts consists not only of mesenchymal cells, but also of collagens and fibronectin [14]. That may explain why we did not find a correlation between interstitial fibrosis and the percentage of vimentin-positive cells although the number of those cells was the highest in the DCM group.

The periostin showed variability in the expression pattern. It changed from a cytoplasmic intra-cardiomyocyte expression in the control group to an intense expression in the interstitial tissue with only mild expression in the cardiomyocyte cytoplasm present in the DCM and MMVD group. Periostin undergoes upregulation in injured and remodelling tissues (including myocardium), [15, 17, 28]. It binds to the extracellular matrix proteins and does not play a direct structural role. Instead, it modulates the cell phenotype and function. It activates cardiac fibroblasts, inducing their migration and transdifferentiation [15]. According to Zhao et al. [28], the increase in interstitial periostin expression shows a high correlation to heart fibrosis. We evaluated both interstitial and cardiomyocyte periostin expression and found no such relationship. However, both greater interstitial fibrosis and more pronounced change in the periostin expression pattern were found in the DCM group than in the MMVD group.

The lower expression of caspase-3 noted in the DCM group as compared to both the MMVD and the control group contradicts the results of other authors. An increased expression of caspsase-3 genes was noted in tachycardia-induced heart failure [29–31]. This may suggest that in DCM, frequently associated with tachyarrhythmias, apoptosis plays a key role in myocardial failure. However, the caspase-3 antibody used in our study was not specific for the inactive and active protein. That finding, as well as the marked caspase-3 expression in the control group, changes in nuclei structure in heart failure, alterations in transcription noted by other authors [26], and the negative correlation between caspase-3 expression and cardiomyocyte degeneration, all suggest that the transcription of caspase-3 may be altered in DCM patients, thus leading to the use of all the available cytoplasmic caspase-3. In other studies of overexpression of caspase-3 in tachycardia-induced cardiomyopathy, the animals usually underwent a post-mortem examination after several weeks of the experiment [29–31]. The dogs in our study presented with stage D heart failure resulting from heart disease that lasted months or years. That may have contributed to the depletion of the available cytoplasmic proteins. On the other hand, Schaper et al. [26] hypothesise that apoptosis plays a minor role in heart failure.

Although the alterations in the expression of the examined proteins were noted in both groups, they were more pronounced in the DCM group than in the MMVD group. In our previous research [10], we noted that the atrial specimens from DCM dogs contained more marked interstitial fibrosis and degeneration of cardiomyocytes, as well as less pronounced perivascular fibrosis and arterial narrowing compared to the MMVD dogs. That finding, together with a lower expression of desmin, vimentin, periostin and caspase-3 in the DCM group than in the control and MMVD groups, explains the negative correlation of the expression of those proteins and the features of cardiomyocyte degeneration (enlargement of the nuclei, loss of striation and structural changes) or perivascular fibrosis. It also explains the positive correlation between the expression of the markers and arterial narrowing.

Owing to the fact that the DCM and MMVD groups did not show significant differences in the LA enlargement (no difference in the LA/Ao ratio between groups), the significant differences in heart remodelling, including differences in the expression of cell markers, indicate that volume overload is not the only factor contributing to myocardial remodelling in DCM. This may suggest a potential role of sustained rhythm disturbances (frequent in DCM and present in all of the examined cases in the DCM group) or primary myocardial dysfunction leading to chamber dilation (as in the ventricular myocardium in dogs with DCM) in the remodelling of the LA in this disease [5, 6].

The examined groups showed significant differences in the expression of the examined proteins. Hence, further studies will be focused on carrying out an analysis of numerous cell markers in both cardiomyocytes and interstitial tissue.

## Conclusion

The expression of desmin, vimentin, periostin and caspase-3 in the left atrium changed in both DCM and MMVD compared to the normal myocardium. The alterations were more pronounced in dogs with DCM than in those with MMVD, and there was no difference in left atrial enlargement between the two groups. This suggests that although both diseases lead to similar left atrial enlargement, the mechanism of remodelling of LA myocardium varies between DCM and MMVD and may be not only a result of that enlargement resulting from volume overload.

## Abbreviations

Ao: Aorta
DCM: Dilated cardiomyopathy
FS: Fractional shortening
LA: Left atrium
LA/Ao: Left atrial-to-aortic root diameter ratio
MMVD: Myxomatous mitral valve disease

## References

[1] Alroy J, Rush JE, Freeman L, Amarendhra Kumar MS, Karuri A, Chase K (2000). Inherited infantile dilated cardiomyopathy in dogs: genetic, clinical, biochemical, and morphologic findings. Am J Med Gen.

[2] Dambach DM, Lannon A, Sleeper MM, Buchanan J (1999). Familial dilated cardiomyopathy of young Portuguese Water Dogs. J Vet Intern Med.

[3] Dukes-McEwan J, Borgarelli M, Tidholm A, Vollmar AC, Häggström J (2003). Proposed guidelines for the diagnosis of canine idiopathic dilated cardiomyopathy. J Vet Cardiol.

[4] Lobo L, Carvalheira J, Canada N, Bussadori C, Gomes JL, Faustino AMR (2010). Histologic characterization of dilated cardiomyopathy in Estrela Mountain Dogs. Vet Pathol.

[5] Tidholm A, Häggström J, Borgarelli M, Tarducci A (2001). Canine idiopathic dilated cardiomyopathy. Part I: aetiology, clinical characteristics, epidemiology and pathology. Vet J.

[6] Tidholm A, Jönsson L (2005). Histologic characterization of canine dilated cardiomyopathy. Vet Pathol.

[7] Häggström J, Duelund Pedersen H, Kvart C (2004). New insights into degenerative mitral valve disease in dogs. Vet Clin North Am Small Anim Pract.

[8] Hanna N, Cardin S, Leung T-K, Nattel S (2004). Differences in atrial versus ventricular remodelling in dogs with ventricular tachypacing-induced congestive heart failure. Cardiovasc Res.

[9] Janus I, Nowak M, Madej JA (2015). Pathomorphological changes of the myocardium in canine dilated cardiomyopathy. Bull Vet Inst Pulawy.

[10] Janus I, Noszczyk Nowak A, Nowak M, Ciaputa R, Kandefer-Gola M, Paslawska U (2016). A comparison of the histopathologic pattern of the left atrium in canine dilated cardiomyopathy and chronic mitral valve disease. BMC Vet Res.

[11] Gofflot S, Kischel P, Thielen C, Radermacher V, Boniver J, De Leval L (2008). Characterisation of an antibody panel for immunohistochemical analysis of canine muscle cells. Vet Immunol Immunop.

[12] Hein S, Kostin S, Heling A, Maeno Y, Schaper J (2000). The role of cytoskeleton in heart failure. Cardiovasc Res.

[13] Pawlak A, Gil RJ (2007). Desmin – an important structural protein of a cardiac myocyte. Kadiol Pol.

[14] Sharov VG, Kostin S, Todor A, Schaper J, Sabbath HN (2005). Expression of cytoskeletal, linkage and extracellular proteins in failing dog myocardium. Heart Fail Rev.

[15] Frangogiannis NG (2012). Matricellular proteins in cardiac adaptation and disease. Physiol Rev.

[16] Katsuragi N, Morishita R, Nakamura N, Ochiai T, Taniyama Y, Hasegawa Y (2004). Periostin as a novel factor responsible for ventricular dilation. Circulation.

[17] Litvin J, Blagg A, Mu A, Matiwala S, Montgomery M, Berretta R (2006). Periostin and periostin-like factor in the human heart: possible therapeutic targets. Cardiovasc Pathol.

[18] Stansfield WE, Andersen NM, Tang RH, Selzman CH (2009). Periostin is a novel factor in cardiac remodeling after experimental and clinical unloading of the failing heart. Ann Thorac Surg.

[19] Regula KM, Kirshenbaum LA (2005). Apoptosis of ventricular myocytes: a means to an end. J Mol Cell Cardiol.

[20] Budihardjo I, Oliver H, Lutter M, Luo X, Wang X (1999). Biochemical pathways of caspase activation during apoptosis. Annu Rev Cell Dev Biol.

[21] Mughal W, Dhingra R, Kirchenbaum LA (2012). Striking a balance: autophagy, apoptosis, and necrosis in a normal and failing heart. Curr Hypotens Rep.

[22] Borgarelli M, Savarino P, Crosara S, Santilli RA, Chiavegato D, Poggi M (2008). Survival characteristics and prognostic variables of dogs with mitral regurgitation attributable to myxomatous valve disease. J Vet Intern Med.

[23] Whitney JC (1967). Cardiovascular pathology. J Small Anim Pract.

[24] Atkins C, Bonagura J, Ettinger S, Fox P, Gordon S, Häggström J (2009). Guidelines for the diagnosis and treatment of canine chronic valvular heart disease. J Vet Intern Med.

[25] Falk T, Jönsson L, Olsen LH, Pedersen HD (2006). Arteriosclerotic changes in the myocardium, lung and kidney in dogs with chronic congestive heart failure and myxomatous mitral valve disease. Cardiovasc Pathol.

[26] Schaper J, Kostin S, Hein S, Elsässer A, Arnon E, Zimmermann R (2002). Structural remodelling in heart failure. Exp Clin Cardiol.

[27] Remmele W, Stegner HE (1987). Recommendation for uniform definition of an immunoreactive score (IRS) for immunohistochemical estrogen receptor detection (ER-ICA) in breast cancer tissue. Pathologe.

[28] Zhao S, Wu H, Xia W, Chen X, Zhu S, Zhang S (2014). Periostin expression is upregulated and associated with myocardial fibrosis in human failing hearts. J Cardiol.

[29] Mahmoudabady M, Niazmand S, Shafei MN, McEntee K (2013). Investigation of apoptosis in a canine model of chronic heart failure induced by tachycardia. Acta Physiol Hung.

[30] Moe GW, Naik G, Konig A, Lu X, Feng Q (2002). Early and persistent activation of myocardial apoptosis, *bax* and caspases: insights into mechanisms of progression of heart failure. Pathophysiology.

[31] Heinke MY, Yao M, Chang D, Einstein R, Dos Remedios CG (2001). Apoptosis of ventricular and atrial myocytes from pacing-induced canine heart failure. Cardiovasc Res.

[32] The Polish Parliament: Act on the protection of animals used for scientific or educational purposes. http://orka.sejm.gov.pl/proc7.nsf/ustawy/2709_u.htm (2015). Accessed 14 Sept 2016.

